# Clinical evaluation of 3D printing and tissue engineering in craniofacial reconstruction: A prospective observational study

**DOI:** 10.6026/973206300212909

**Published:** 2025-08-31

**Authors:** Subramanian P., Timbadiya Vijaykumar Mansukhbhai, Parth Shailesh Bhatt, Shivalika Bhatnagar, Rashmi Laddha, Rishi Desai

**Affiliations:** 1 Department of Oral and Maxillofacial Surgery, Dhanalakshmi Srinivasan Dental College, Perambalur, Tamil Nadu, India; 2 Department of Oral Pathology and Microbiology, Rishiraj College of Dental Science and Research Centre Bhopal, Madhya Pradesh, India; 3 Department of Pathology, Dr. N.D. Desai Faculty of Medical Science & Research, Dharmsinh Desai University, Nadiad, Gujarat, India; 4 Department of Dentistry, DY Patil University School of Dentistry, Nerul, Navi Mumbai, Maharashtra, India; 5 Department of periodontology Dr RR Kambe Dental college and hospital Kanheri, Akola, Maharashtra, India

**Keywords:** Craniofacial reconstruction, 3D printing, tissue engineering, patient-specific implants, prosthetics, regenerative medicine, biocompatibility

## Abstract

The clinical results of tissue engineering and 3D printing in craniofacial reconstruction in 60 patients followed up over 3 years. Patient-specific implants and tissue-engineered scaffolds were created with state-of-the-art 3D printing techniques together with autologous stem cells and growth factors. Excellent success rates were attained with 92% implant integration, 88% satisfaction with aesthetics, and 85% return to function, with complications being restricted to 10% and treated conservatively. The technique decreased operative time, hospital stay and total cost of treatment substantially when compared with traditional techniques. Thus, we show the clinical application of 3D printing and tissue engineering as an effective, safe, and economically viable method in craniofacial reconstructive surgery.

## Background:

The rising incidence of defects in oral and maxillofacial tissues, linked to factors such as trauma, tumors, periodontal disease, and aging, poses significant challenges. Current treatments, involving autografts, allografts, and synthetic graft materials, face obstacles like secondary trauma, inflammation, and inadequate biocompatibility [1]. Anatomical complications of the craniofacial regions often present considerable challenges to the surgical repair or replacement of the damaged tissues [2]. Three-dimensional (3D) printing has been particularly widely adopted in medical fields. Application of the 3D printing technique has even been extended to bio-cell printing for 3D tissue/organ development, the creation of scaffolds for tissue engineering, and actual clinical application for various medical parts [3]. Three-dimensional (3D) bioprinting technology will play a pivotal role in medicine, offering a promising potential for bone reconstruction, rehabilitation and regeneration and expanding treatment options in many fields of operation [4]. Craniofacial reconstruction is one of the most challenging problems in contemporary reconstructive surgery because of the delicate anatomy, the functional importance and the esthetic sensitivity of the craniofacial complex. Traditional surgical methods like autologous bone transplantation, prosthetic implants and soft tissue transfer have been used for decades but have major drawbacks [5]. These are donor site morbidity, infection risk and long operative time, staged multiple procedures and compromised cosmetic results [6]. Moreover, the failure to accurately reproduce the intricate three-dimensional craniofacial architecture further undermines surgical outcomes. Recent developments in 3D printing technology and tissue engineering have transformed craniofacial surgery with the use of highly customized, patient-specific treatments [7]. 3D printing or additive manufacturing allows for the direct fabrication of customized implants, prosthetics and surgical guides from high-resolution imaging information [8]. Patient-specific implants enable accurate anatomical reconstruction, minimizing intraoperative changes, surgical duration and related complications [9]. In addition, the mechanical properties of 3D printed tissue can be designed to closely replicate native bone and soft tissues. Tissue engineering synergizes with 3D printing by allowing regeneration of biological tissue through the use of a combination of biocompatible scaffolds, stem cells and growth factors [10]. This technology has the potential to regrow bone, cartilage and soft tissues with enhanced biological integration, minimizing dependence upon synthetic materials and the risk of immune rejection [11]. The combination of these technologies presents a promising avenue for enabling superior functional and aesthetic results in craniofacial reconstruction [12]. Even with these technological innovations, issues of vascularization of engineered tissues, long-term implant stability, biocompatibility and cost remain as impediments to their broad clinical use [13]. In addition, although numerous experimental studies and case reports have shown encouraging outcomes, standardized clinical testing is needed to validate the efficacy, safety and long-term results of these new reconstructive modalities [14]. Therefore, it is of interest to assess the results, complications and cost-effectiveness of 3D printing and tissue engineering in craniofacial reconstruction in a prospective patient cohort, with the objective to provide evidence-based real-world clinical evidence to substantiate routine use of these technologies in reconstructive surgery.

## Materials and Methods:

This prospective observational study was conducted over a period of three years from January 2021 to December 2023. A total of 60 patients aged between 18 and 65 years, diagnosed with craniofacial defects of either congenital or acquired origin, requiring surgical reconstruction, were enrolled in the study after obtaining written informed consent. Exclusion criteria included patients with severe systemic disease, active infections, noncompliance with follow-up, or prior failed craniofacial reconstructive surgery. All patients were scanned with high-resolution computed tomography (CT) and had the DICOM data treated with CAD software to create patient-specific three-dimensional models. Tailored implants and scaffolds were created with the aid of superior 3D printing technologies using titanium alloy (Ti-6Al-4V) for hard skeletal reconstruction by selective laser melting (SLM) and biocompatible polylactic acid (PLA) with hydroxyapatite (HA) scaffolds fabricated through fused deposition modeling (FDM) for tissue engineering purposes. In applicable cases, autologous bone marrow-derived mesenchymal stem cells (BM-MSCs) combined with platelet-rich plasma (PRP) were seeded onto scaffolds to promote osteogenesis and soft tissue regeneration. All surgical procedures were performed under general anesthesia by the same surgical team for consistency, with intraoperative adjustments minimized due to the precise fit of the customized implants. Postoperative assessments were conducted at 1, 3, 6, and 12 months, evaluating implant integration, functional restoration (including mastication, speech, and occlusion), aesthetic outcomes through both patient satisfaction and surgeon evaluation, postoperative complications, operative time, hospital stay, and cost analysis.

## Results:

A total of 60 patients were included in the study conducted over three years. The majority of cases involved post-traumatic craniofacial defects, followed by congenital deformities, post-tumor resection, and facial asymmetry corrections. Customized 3D-printed implants fabricated from titanium, polylactic acid, and composite materials were successfully utilized, with tissue-engineered scaffolds integrated in selected cases using stem cells and platelet-rich plasma. The overall surgical success rate was 91.7%, with significant functional recovery (85%) and excellent to good aesthetic outcomes achieved in 88.3% of cases. The postoperative complication rate was low (10%), while 3D printing significantly reduced operative time, hospital stay, and treatment cost compared to conventional reconstruction methods, with high patient satisfaction observed at follow-up. Table 1 (see PDF) shows the distribution of study subjects based on the type of craniofacial defect. Post-traumatic defects were the most common indication for reconstruction, followed by congenital deformities and post-tumor resection defects. Table 2 (see PDF) shows the distribution of patients based on age groups. The majority of the patients belonged to the age group of 31-40 years. Table 3 (see PDF) shows the gender distribution of the study population. A higher proportion of males underwent craniofacial reconstruction compared to females. Table 4 (see PDF) shows the types of 3D-printed implants used. Titanium implants were used in the majority of cases. Table 5 (see PDF) shows the use of tissue engineering components integrated with 3D-printed scaffolds. Stem cell-based scaffolds were applied in over half of the cases. Table 6 (see PDF) shows the overall surgical success rates observed in the study, with high rates of implant stability and successful integration. Table 7 (see PDF) shows the functional recovery outcomes assessed at 12-month follow-up. Table 8 (see PDF) shows the aesthetic outcome based on patient and surgeon evaluation. Table 9 (see PDF) shows the observed postoperative complications. Table 10 (see PDF) shows and compares the average operative time and hospital stay between 3D printing-based reconstruction and conventional reconstruction approaches. Table 11 (see PDF) shows and compares the overall cost of reconstruction using 3D printing technology versus conventional techniques. Table 12 (see PDF) shows patient satisfaction scores evaluated using a 10-point Likert scale.

## Discussion:

Craniofacial reconstruction remains one of the most technically challenging fields in maxillofacial and reconstructive surgery due to the complex anatomy, critical functional roles, and high aesthetic demands associated with the craniofacial region [15]. The conventional reconstructive approaches often involve autologous bone grafting, alloplastic implants, soft tissue flaps, or a combination of these, each associated with its own limitations including donor site morbidity, prolonged operative time, multiple surgeries, and suboptimal functional or cosmetic outcomes [16]. The emergence of 3D printing and tissue engineering technologies has introduced new possibilities that offer improved precision, customization, and clinical outcomes while addressing many of the shortcomings of traditional methods [17]. The application of 3D printing allows the creation of patient-specific implants designed precisely according to the individual's anatomy, using digital imaging and computer-aided design [18]. This result in superior anatomical fit, reduced intraoperative modifications, and improved functional outcomes. Additionally, the shorter operative time and intraoperative manipulations of pre-fabricated custom implants play an important role in the general safety and economy of the procedure [19]. The presence of titanium and hybrid composite materials showed substantial mechanical stability and strength, in accordance with biomechanical properties previously described for 3D-printed prostheses to be applied to craniofacial reconstruction [20]. Concurrently, tissue engineering provides additional advantages by promoting the biological regeneration of hard and soft tissues using biocompatible scaffolds seeded with stem cells and bioactive factors [21]. The combination of bone marrow-derived mesenchymal stem cells with platelet-rich plasma in carefully chosen patients helped improve osteogenesis and healing of soft tissues, diminishing the use of foreign implants and improving the long-term biological integration of the reconstructed tissues [22]. These methods as a whole represent a potential for more physiological and more lasting reconstruction with more complete restoration of function and appearance [23]. In spite of these benefits, the clinical application of 3D printing and tissue engineering is not free from challenges. The fabrication process involves dedicated equipment, sophisticated imaging, software skills, and multidisciplinary input, which could make it unavailable widely, particularly in resource-poor environments [24. Additionally, although short-term results are encouraging, long-term results assessing the stability, wear characteristics, and biocompatibility of these individually tailored implants are limited and require ongoing longitudinal studies. Vascularization is yet another vital limitation in bigger tissue-engineered constructs, which has direct impacts on the grafts' viability and integration, especially in large or complex craniofacial defects. However, the cost-benefit analysis noted herein reinforces the economic feasibility of 3D printing and tissue engineering over traditional surgical methods. Decreased operative time, hospital admission, and total treatment expense, along with greater aesthetic and functional results, highlights the clinical applicability of integrating these newer technologies into standard reconstructive practice. The current study affirms the expanding evidence base that supports 3D printing and tissue engineering as safe, effective, and highly efficient modalities of craniofacial reconstruction with better outcomes and fewer complications. Further research aimed at overcoming the current technical and biological limitations, especially vascularization and long-term implant stability, will continue to expand the range and applicability of these technologies in reconstructive surgery.

## Conclusion:

The combination of 3D printing and tissue engineering in craniofacial reconstruction has important benefits in terms of surgical accuracy, functional recovery, and cosmetic appearance with few complications. These technologies had excellent surgical success and patient satisfaction and lowered operative time, hospital stay, and overall cost of treatment. Additional clinical studies aimed at long-term stability and vascularization can further improve the outcomes and expand the scope of these applications.
